# Maximizing the Field Emission Performance of Graphene Arrays

**DOI:** 10.3390/nano10102003

**Published:** 2020-10-11

**Authors:** Kaiqiang Yang, Jianlong Liu, Ruirui Jiang, Yubing Gong, Baoqing Zeng, Jianjun Yang, Feng Chi, Liming Liu

**Affiliations:** 1 School of Electronic Science and Engineering, University of Electronic Science and Technology of China, Chengdu 610054, China; 201811022515@std.uestc.edu.cn (K.Y.); liujianlong@uestc.edu.cn (J.L.); 201711040120@std.uestc.edu.cn (R.J.); ybgong@uestc.edu.cn (Y.G.); 2 Zhongshan Branch of State Key Laboratory of Electronic Thin Films and Integrated Devices, University of Electronic Science and Technology of China, Zhongshan Institute, Zhongshan 528402, China; sdyman@uestc.edu.cn (J.Y.); chifeng@semi.ac.cn (F.C.)

**Keywords:** graphene, field emission, field screening effect, cold cathode

## Abstract

To design efficient and powerful field emission cathodes, the screening effect is of great importance and should be traded off between screening and emitter number. It has long been found that to achieve maximum emission efficiency in an array, neighboring emitters are at two or three times their height from each other. However, this is only true for one-dimensional emitters, such as carbon nanotubes, but for graphene, a two-dimensional material, it is different. In this work, we found that to achieve maximum emission efficiency in an array of graphene, the separation of the emitter is four times the height, and it is insensitive to the anode voltage and the distance between the cathode and the anode.

## 1. Introduction

Field emitter arrays (FEAs) have been an active area of research for many years due to the high current densities, low voltage operation, and high pulse repetition that can be obtained [1,2,3,4,5,6]. The proximity of emitters in an array is well known to lead to electric field screening (EFS), which makes the emission currents from the tips not uniform and, in turn, affects the amount of current that can be liberated from FEAs. Therefore, investigating EFS of periodic FEAs is of great importance, especially for the design of efficient and powerful multi-emitter devices [7,8,9,10,11,12,13,14,15,16].

Although much research has been done on the EFS, most of it has been based on the EFS from carbon nanotubes (CNT) arrays in simulations. For example, in 2000, Nilsson et al. found that an optimum intertube distance of two times the height of the CNT is strongest in accordance with the experiment [17]. However, in 2009, Smith et al. found that the previous optimum separation of twice the height was an under-estimate of screening in 3D, and to achieve a fully unscreened array, the optimum spacing needs to be in excess of three times the height of the CNT [18].

Graphene is a monolayer of carbon atoms packed into a dense honeycomb crystal structure that can be viewed as an individual atomic plane extracted from graphite [19]. The atomic thickness, high aspect ratio, excellent electrical conductivity [20,21], and good mechanical properties make graphene a competitive candidate for field emission applications [22,23]. Although a great deal of research on field emission of graphene arrays has been reported [24,25,26,27,28,29], currently, graphene sheets in arrays are generally stacked into pieces and distributed randomly due to the limitation of the current preparation methods, the poor orientation, and the messy distribution of graphene sheets in arrays produce an electric field screening effect, which reduces the electric field strength of edges of the graphene sheets and inhibits the field emission performance. Therefore, studying the reasonable arrangement of graphene sheets in the arrays has become our current work.

In this paper, we simulated the field screening effect in graphene arrays, and the effects of various parameters in the model on the field screening effect were investigated in detail. We also compared the simulated current density of the graphene sheet array that we optimized with some previous experimental results about field emission of graphene arrays prepared by different methods.

## 2. Physical Model

**Figure 1:**
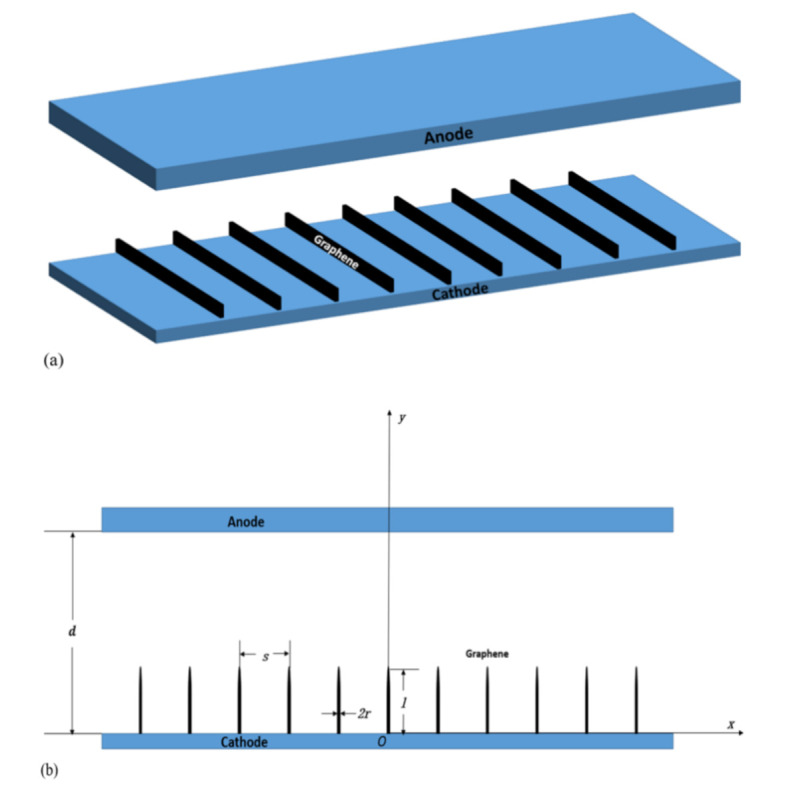
Structure of graphene array, (**a**) is a 3D image of the graphene array, (**b**) is the main view of the field emission structure of the graphene sheet arrays.

Figure 1a shows a 3D image of the graphene array. Figure 1b shows a schematic of the simulated array of graphene. Vertically aligned graphene of uniform height *l* and thickness 2*r* are placed on a grounded cathode that is separated from the positive anode by distance *d*; the graphene is separated by a distance *s*, the anode voltage is *U*. Assuming that the thickness and height of the graphene in the array are the same and the top of the graphene sheet is assumed to be circular. The emitted current depends on the electric field in the vicinity of the graphene top. The emission array performance was studied by simulating a unit cell consisting of the integrated flake nanostructure and the anode.

## 3. Simulation Method

**Figure 2:**
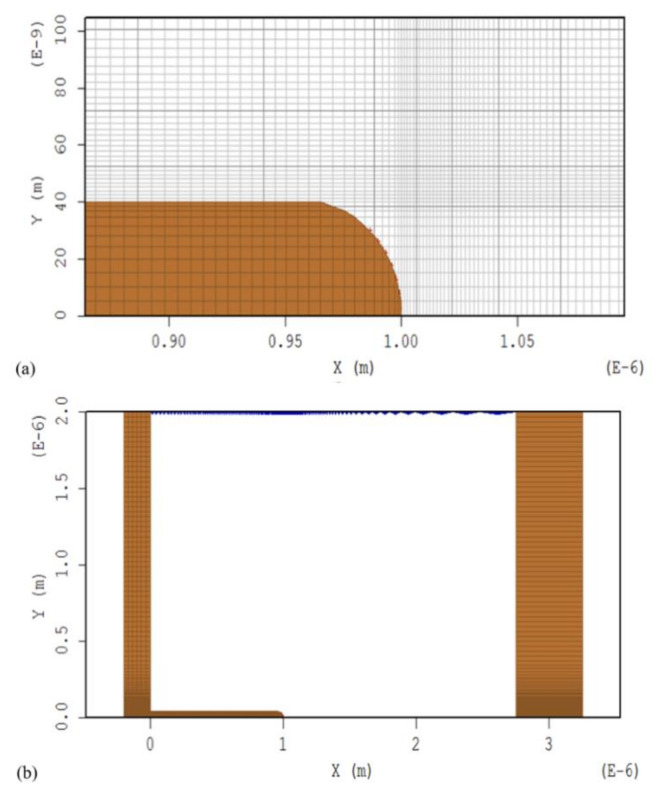
Simulation model of the graphite nanoplatelet array in MAGIC. (**a**) shows the mesh distribution of the emitter tip in MAGIC; (**b**) is one unit of the graphite nanoplatelet array in MAGIC.

Because the radius of the curvature of the tip of the emitter is very small on the nanometer scale, and the device structure is relatively large, the skills of variable mesh are used. The accurate field strength at the tip of the emitter is obtained from denser grids, and sparse grids are used far from the emitter to speed up the program. Figure 2a shows the mesh distribution of the emitter tip in MAGIC. To further simplify this model, we used a two-dimensional model to replace the 3D model, which is symmetrical with this two-dimensional plane. This two-dimensional model is located on the XOY plane. In the simulation, MAGIC will default to give this 2D model 1 m extension in the Z direction, so that the actual simulation is 3D.

Calculations were performed using the MAGIC software code. MAGIC is a particle-in-cell simulation code, which is a two-dimensional, finite-difference, time-domain code for the self-consistent simulation of the interaction between charged particles and electromagnetic fields. The full set of Maxwell equations and the complete Lorentz force equation were used. Space charge effects were automatically included. Boundary conditions included conductors, the surface of graphite nanoplatelets, and the cell-to-cell relationships using SYMMETRY MIRROR boundary conditions, which allowed us to simulate an entire array, not just a cell as in many previous works. Figure 2b shows one unit of the graphite nanoplatelet array in MAGIC. The two-dimensional model in Figure 2 can be copied by the mirror boundary conditions of the lower boundary, and the copied model can be copied by the mirror boundary conditions of the upper boundary again. Thus, an extended wireless array can be obtained [30,31].

A Fowler-Nordheim emission module was developed and inserted in MAGIC code to model the electron field emission from the surface. The current density *J* is calculated from

$$J=\frac{AE_{local}^{2}}{\varphi t^{2}\left(y\right)}\exp\left(\frac{-B\nu \left(y\right)\varphi^{\frac{3}{2}}}{E_{local}}\right)$$

where $A=1.5414\times 10^{-6}\;A/m$, $B=6.8308\times 10^{9}\;m^{-1}V^{-1/2}$, $E_{local}$ are the normal components of the field at the emitter surface and φ is the work function of the graphene. In this paper, we have taken 5.0 eV for graphene, $t^{2}\left(y\right)=1.1$, an approximation, and $\nu \left(y\right)=0.96-y^{2}$ with $y=3.79\times 10^{-5}\times E^{1/2}/h$ in SI units [32]. Initially, the Green’s function solutions of the Laplace equation were computed for the given geometry. The electric field along the emitter surface was determined by the superposition of the contribution from each conductor. The emitted charge is governed by the Fowler-Nordheim relation applied locally at each cell along the emitter. The electric field at the emitter surface was then adjusted by Gauss’s law to allow for field perturbation due to the emitted charge. The simulation proceeded, after running this module, by propagating the particles, updating the fields, and then self-consistently evolving in time to determine the new emitted current required, using the updated fields. This process continued for the specified number of loops. In the simulation, we defined normalized spacing as *s*/*l*.

## 4. Results and Discussion

Although the skill of variable grid is used to achieve simulation of the field emission characteristics of emitters with nanometer-scale thickness, because the thickness of graphene is an atomic scale and the total number of grids that MAGIC can draw is limited, even the skill of variable grid cannot simulate the electric field at the top of the emitter of the graphene sheet. Therefore, we firstly studied the effect of the thickness of graphite nanoplatelets to the screening effects; graphite nanoplatelet is a kind of graphite sheet with nanometer thickness, graphene is actually a graphite sheet with only one or more atomic layers. In the simulation, we changed the spacings between the graphite nanoplatelets in arrays with different thicknesses. 

**Figure 3:**
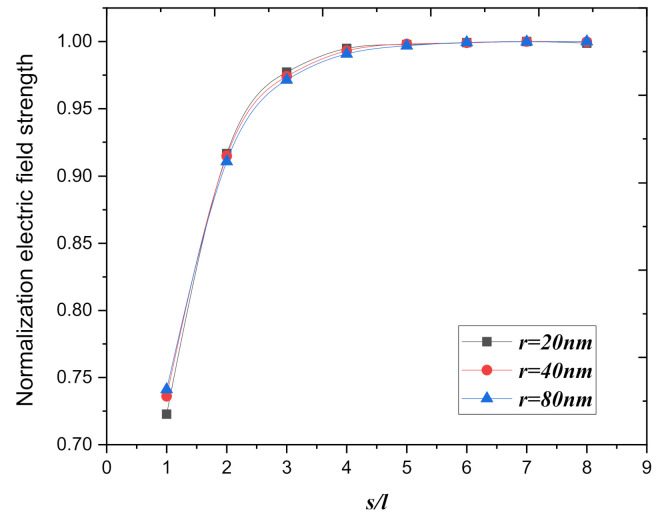
The normalization of the electric field strength of the graphite nanoplatelet in the middle of the array as a function of the normalization spacing of *s/l*, the thickness of the nano graphite sheet is chosen to be 20 nm, 40 nm, 80 nm. (*U* = 200 V, *d* = 2.75 μm).

Figure 3 shows that the normalization of the electric field strength of the graphite nanoplatelet in the middle of the array as a function of *s*/*l*. The result shows that the law of change of electric field strength at the tip of the graphite nanoplatelet in the middle of the array caused by the field screening effect is insensitive to the thickness of the graphite nanoplatelets; the three curves are approximately coincident. All show that the electric field strength at the top of the emitter increases with the increase of the spacing of the array. For *s*/*l* > 5, the value of the normalization of the electric field strength reaches saturation. So, we focused on studying the effect of different spacings between the graphite nanoplatelets and the screening effects of graphite nanoplatelets arrays. Simulation with different thicknesses of graphite nanosheet arrays had almost no effect on the results; thus we used graphite nanosheets (about 40 nm thickness, *r* = 20 nm) instead of graphene to simulate.

**Figure 4:**
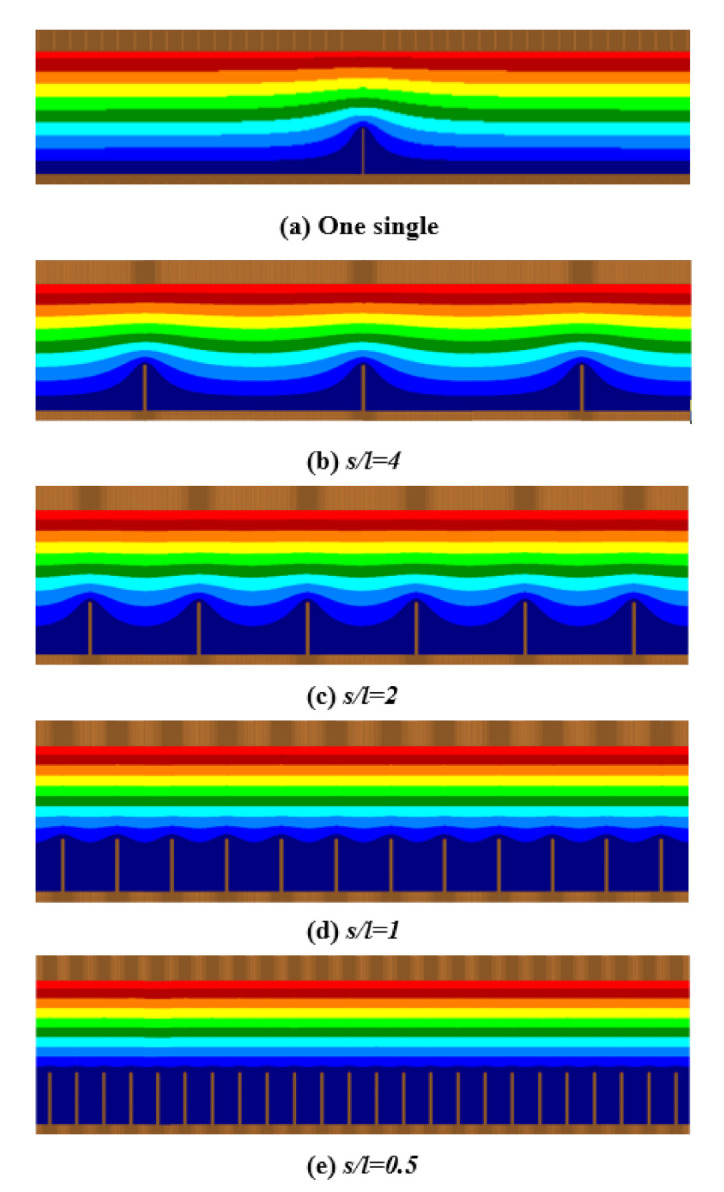
The potential distribution of graphite sheet arrays with a thickness of 40 nm at field emission (**a**): single root, (**b**): *s/l* = 4, (**c**): *s/l* = 2, (**d**): *s/l* = 1, and (**e**): *s/l* = 0.5.

We simulated the potential distribution of single platelets and graphene arrays with different spacings; the anode voltage was 400 V. Figure 4 shows that with the decrease of spacing between graphene sheets in the array, the screening effects of the graphene array becomes stronger, as expected. When *s*/*l* = 0.5, there is almost no potential distribution around the top of the emitter. When *s*/*l* = 4, the interaction between emitters is very small, and the electric potential distribution is similar to that of a single emitter.

**Figure 5:**
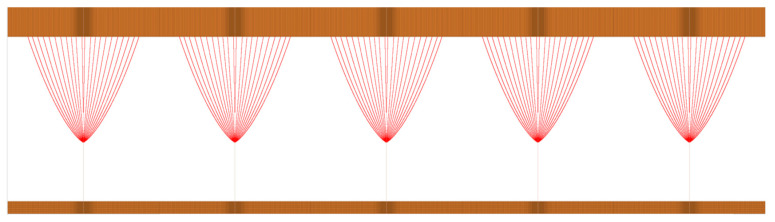
Electron beam trajectory of the field emission from a graphite sheet array (*s/l* = 4, *U* = 200 V, and *d* = 2.75 μm).

Because the field emission current is exponentially related to the electric field intensity at the top of the emitter, the emission current of the emitter at the center of the array is also simulated by a particle simulation method. Figure 5 shows the electron beam trajectory of the field emission from a graphite nanoplatelet array; the parameters are as follows: *s*/*l* = 4 and *U* = 200 V.

**Figure 6:**
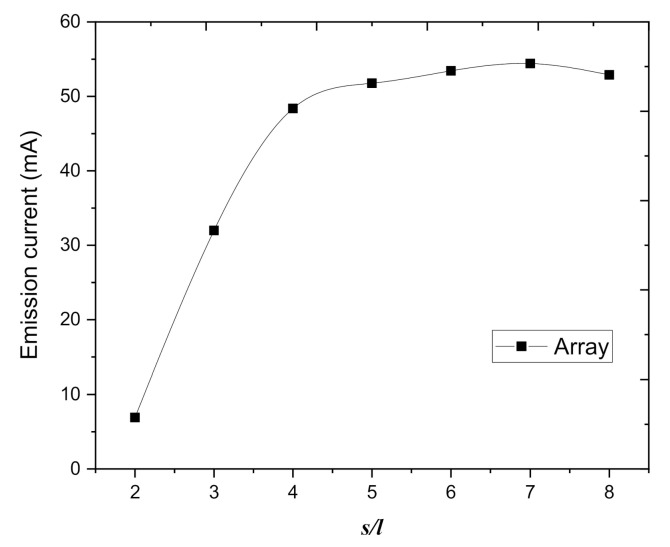
The relation between normalized spacing and the field emission current of the graphite sheet at the center of the array. (*U* = 200 V, *d* = 2.75 μm).

Figure 6 shows the current of graphite nanoplatelets in the middle of the array as a function of the spacing between the graphite nanoplatelets. In the simulation, the parameters were as follows: *U* = 400 V and *r* = 20 nm. The current of graphite nanoplatelets in the middle of the array increases with the increase of spacing between graphite nanoplatelets; for *s/l >* 5, the value of current of graphite nanoplatelets in the middle of the array reaches saturation. The result demonstrates that with the increase of the spacing between graphite nanoplatelets, the screening effects decrease, the electric field strength increases, the current of graphite nanoplatelets in the array tends to be saturated, and the behavior of the individual graphite nanoplatelet is similar to that of a single one. Therefore, we can weaken the screening effects and increase the current of each emitter of the array by changing the spacing between emitters in the array.

Although the emission of a single emitter is not affected by the screening effects, the current of a single emitter is limited, which cannot meet the requirements of the device. According to the above simulated calculation, for graphene arrays, the screening effects can be neglected when the spacing between emitters is five times higher; on the other hand, due to the enlargement of the spacing between emitters, the number of emitters on a fixed area decreases and the total current on the surface decreases. Because the spacing between emitters is very close, the screening effects will affect the value of the current of each emitter in the array, which makes the total emitter current smaller. Therefore, it is necessary to find the appropriate array density by simulated calculation.

So, we performed further simulation for a total array width of 1 mm. Because the default *Z*-axis depth is 1 m in the MAGIC simulation software, the actual fixed area obtained is 10 square centimeters. We assumed that the current of each graphite nanoplatelet in the array is equal. When the spacing of graphite nanoplatelets in the array is twice the height of the emitter, the number of emitters on the area is 500, when the spacing of graphite nanoplatelets in the array is three times the height of the emitter, the number of emitters on the area is 333, and so on. The number of emitters on the launching surface decreases with the increase of the spacing of emitters, and then the total emission current of all emitters on the launching surface is calculated.

**Figure 7:**
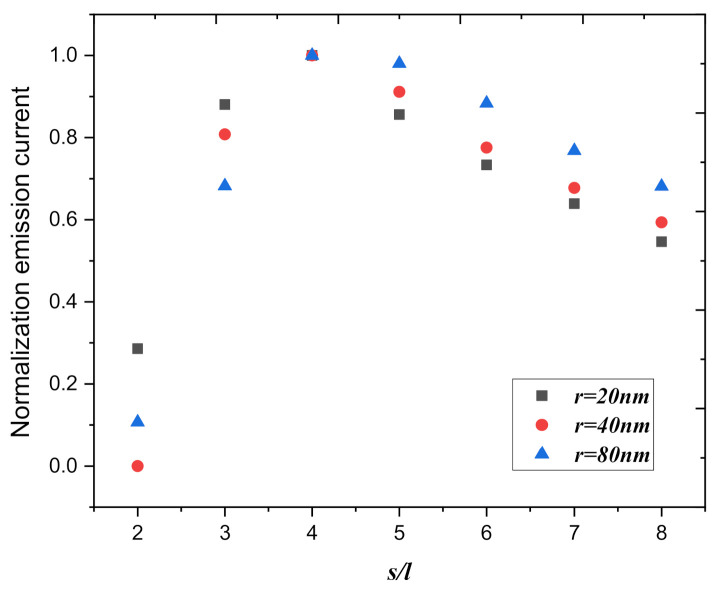
The normalization field emission current in 10 cm^2^ as a function of the normalization spacing, for the aspect *r* (one-half thickness of graphite nanoplatelet), is chosen to be 20 nm, 40 nm, and 80 nm (*U* = 200 V, *d* = 2.75 μm).

Firstly, the anode voltage was 200 V, we used a graphite nanoplatelet array with different thicknesses and changed the spacing between the graphite nanoplatelets in the array, then calculated the total current in this area. Figure 7 shows the field emission current in 10 cm^2^ as a function of the normalization spacing, for the aspect *r* (one-half thickness of graphite nanoplatelet) chosen to be 20 nm, 40 nm, and 80 nm, when the spacing of the graphite nanoplatelet between the array is four times the height of the graphite nanoplatelet, the total emission current of the emitter surface is the largest, and the law is the same for different thicknesses of graphite nanoplatelet. This also corroborates the results of previous simulations that if we focus on studying the effect of different spacings between graphite nanoplatelets and the screening effects of graphite nanoplatelets arrays, the results are insensitive to different thicknesses of graphite nanoplatelets. Therefore, in the later simulation, we still used graphite nanoplatelets with a thickness of 40 nm (*r* = 20 nm), instead of graphene sheets. On a fixed area of the emitter, the number of emitters on the emitter surface is determined by the distance between the emitters. The more the number of emitters on the emitter surface, the greater the emission current on the fixed area. However, the greater the number of emitters, the smaller the distance between emitters, and the more obvious the field screening effect becomes. In order to obtain the maximum field emission current on a fixed area, it is necessary to find a balance between the distance and the number of emitters. *s*/*l* = 4 is the equilibrium in our simulations.

**Figure 8:**
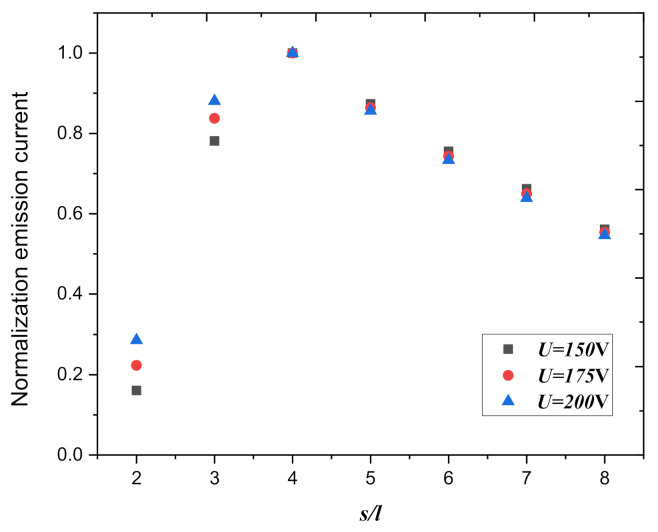
The normalization field emission current in 10 cm^2^ as a function of the normalization spacing, for the anode voltage, is chosen to be 150 V, 175 V, and 200 V (*d* = 2.75 μm).

The thickness of the graphite sheet was 40 nm (*r* = 20 nm); we changed the spacings between the graphite nanoplatelets in the array with different anode voltages and calculated the total emission current of the area. Figure 8 shows that the normalization field emission current in 10 cm^2^ as a function of the normalization spacing, for the anode voltage is chosen to be 150 V, 175 V, and 200 V. When the spacing of the graphite nanoplatelets in the array is four times the height of graphite nanoplatelets, the total emission current of the emitter surface is the largest, and the law is insensitive to anode voltages.

**Figure 9:**
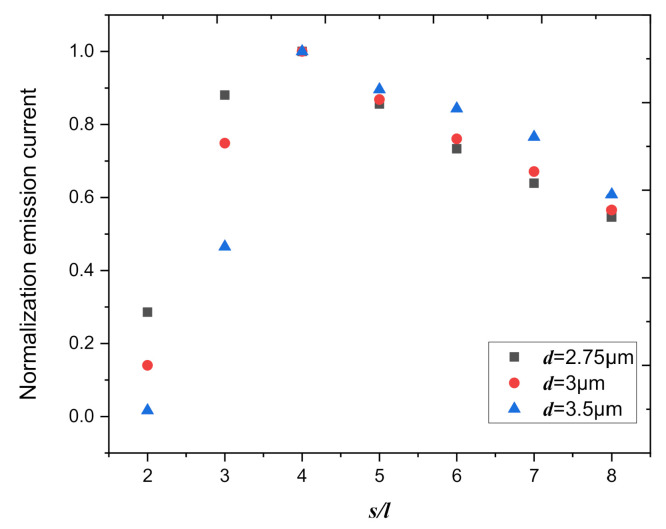
The normalization field emission current in 10 cm^2^ as a function of the normalization spacing, for the distance between cathode and anode, is chosen to be 2.75 μm, 3 μm, and 3.5 μm (*U* = 200 V).

We also calculated the total emission current in this area with different distances between the cathode and anode. Figure 9 shows that the normalization field emission current in 10 cm^2^ as a function of the normalization spacing, for the distance between cathode and anode is chosen to be 2.75 μm, 3 μm, and 3.5 μm. When the spacing of the graphite nanoplatelets in the array is four times the height of graphite nanoplatelets, the total emission current of the emitter surface is the largest, and the law is insensitive to the distance between cathode and anode.

We selected an optimized field emission current density in the simulation to compare with some previous experimental results about field emission of graphene array cathodes. Table 1 lists the field emission current density of graphene array cathodes prepared by different methods. Our optimized simulation results are higher than most of them. The reasonable distribution of graphene sheets in arrays weakens the field screening effect, and the field emission currents of graphene arrays are increased.

## 5. Conclusions

In this paper, calculations were performed using the MAGIC software code, and the technique of variable mesh was used. We focused on the effect of different spacings between graphite nanoplatelets to study the screening effects of graphite nanoplatelets arrays. Simulations show that different thicknesses of graphite nanosheets in arrays have almost no effect on the results. Therefore, we used thicker (40 nm thickness, *r* = 20 nm) graphite nanosheets instead of graphene to simulate. We found that when the spacing between the graphene in the graphene array is five times the height of the graphene, the screening effect is greatly weakened. The current of graphene emitters in the array tends to be saturated, and the behavior of each individual graphite nanoplatelet is similar to that of a single one. We also found that when the spacing between the graphene in the graphene array is 4 times the height of the graphene, the total emission current of the emitter surface is the largest, and the law is insensitive to the anode voltage and the distance between cathode and anode. The emission current density of graphene arrays optimized by our simulation is better than in most previous experimental results about field emission of graphene array cathodes, the results of this simulation can provide guidance for the design of graphene array cathode emitters.

## Figures and Tables

**Table 1:** Field emission current density of graphene array cathodes prepared by different methods.

Methods	Current Density (mA/cm^2^)
Electrophoretic Deposition [23]	0.3
Transferring graphene to ZnO [29]	0.46
Transferring graphene to Nickel Nanotip [28]	1.3
screen-printed [26]	2.6
PECVD [27]	12
**Our optimized simulation result**	**47.88**
MPCVD [25]	105.6
